# Human milk 3’-Sialyllactose is positively associated with language development during infancy

**DOI:** 10.1093/ajcn/nqab103

**Published:** 2021-05-21

**Authors:** Seoyoon Cho, Ziliang Zhu, Tengfei Li, Kristine Baluyot, Brittany R Howell, Heather C Hazlett, Jed T Elison, Jonas Hauser, Norbert Sprenger, Di Wu, Weili Lin

**Affiliations:** Department of Biostatistics, University of North Carolina at Chapel Hill, Chapel Hill, NC, USA; Department of Biostatistics, University of North Carolina at Chapel Hill, Chapel Hill, NC, USA; Department of Radiology, University of North Carolina at Chapel Hill, Chapel Hill, NC, USA; Biomedical Research Imaging Center, University of North Carolina at Chapel Hill, Chapel Hill, NC, USA; Biomedical Research Imaging Center, University of North Carolina at Chapel Hill, Chapel Hill, NC, USA; Fralin Biomedical Research Institute at Virginia Tech Carilion (VTC), Department of Human Development and Family Science, Virginia Polytechnic Institute and State University, Roanoke, VA, USA; Department of Psychiatry, University of North Carolina at Chapel Hill, Chapel Hill, NC, USA; Institute of Child Development, University of Minnesota, Minneapolis, MN, USA; Nestlé Institute of Health Sciences, Société des Produits Nestlé SA, Lausanne, Switzerland; Nestlé Institute of Health Sciences, Société des Produits Nestlé SA, Lausanne, Switzerland; Department of Biostatistics, University of North Carolina at Chapel Hill, Chapel Hill, NC, USA; Division of Oral and Craniofacial Health Science, Adams School of Dentistry, University of North Carolina at Chapel Hill, Chapel Hill, NC, USA; Department of Radiology, University of North Carolina at Chapel Hill, Chapel Hill, NC, USA; Biomedical Research Imaging Center, University of North Carolina at Chapel Hill, Chapel Hill, NC, USA

**Keywords:** human milk, early cognitive development, Mullen Scales of Early Learning, human milk oligosaccharides, sialyllactose, language, random linear mixed effects model, breastfed infants

## Abstract

**Background:**

Genetic polymorphisms leading to variations in human milk oligosaccharide (HMO) composition have been reported. Alpha-Tetrasaccharide (A-tetra), an HMO, has been shown to only be present (>limit of detection; A-tetra+) in the human milk (HM) of women with blood type A, suggesting genetic origins determining the presence or absence (A-tetra-) of A-tetra in HM.

**Objectives:**

This study aimed to determine whether associations exist between HMO concentrations and cognitive development, and whether the associations vary between A-tetra+ and A-tetra- groups in children (<25 months old).

**Methods:**

We enrolled typically developing children (2–25 months old; mean, 10 months old) who were at least partially breastfed at the study visit. The Mullen Scales of Early Learning (MSEL) were used as the primary outcome measure to assess early cognitive development. Linear mixed effects models were employed by stratifying children based on A-tetra levels (A-tetra+ or A-tetra-) to assess associations between age-removed HMO concentrations and both MSEL composite scores and the 5 subdomain scores.

**Results:**

A total of 99 mother-child dyads and 183 HM samples were included (A-tetra+: 57 samples, 33 dyads; A-tetra-: 126 samples, 66 dyads). No significant association was observed between HMOs and MSEL when all samples were analyzed together. The composite score and 3’-sialyllactose (3’-SL) levels were positively associated [*P* = 0.002; effect size (EF), 13.12; 95% CI, 5.36–20.80] in the A-tetra + group. This association was driven by the receptive (adjusted *P* = 0.015; EF, 9.95; 95% CI, 3.91–15.99) and expressive (adjusted *P* = 0.048; EF, 7.53; 95% CI, 2.51–13.79) language subdomain scores. Furthermore, there was an interaction between 3’-SL and age for receptive language (adjusted *P* = 0.03; EF, -14.93; 95% CI, -25.29 to -4.24).

**Conclusions:**

Our study reports the association of 3’-SL and cognition, particularly language functions, in typically developing children who received HM containing detectable A-tetra during infancy.

## Introduction

A wealth of evidence has documented the health benefits of breastfeeding during infancy (1). Human milk (HM) contains a wide array of nutrients and bioactives; among the latter are a vast amount and diversity of human milk oligosaccharides (HMOs) (2, 3). HMOs, the third most abundant solid component of HM, are formed in the mammary glands starting from lactose by the action of a series of glycosyltransferases that elongate lactose with 1 or several moieties of N-acetyl-lactosamine, lacto-N-biose, fucose (Fuc), and sialic acid (4). Several lines of evidence have revealed the potential health benefits of HMOs (3, 5–14). In particular, both sialyllactose (SL) (15–20) and 2’-fucosyllactose (2’-FL) (21–24) have been implicated to benefit early cognitive development in preclinical studies, suggesting that early life dietary supplementation with SL and 2’-FL improved learning ability and spatial cognition and reduced stress-induced anxiety (15–18, 21–23). Furthermore, Berger et al. (24) reported that greater exposure to 2’-FL relative to other HMOs at 1 month but not at 6 months of age was associated with better cognitive development at 24 months. More recently, Oliveros et al. (25) reported a positive association between 1 month postpartum HM 2’-FL levels and 6 month motor scores, whereas 6’-sialyllactose (6’-SL) levels were positively associated with cognitive and motor scores at 18 months.

While the aforementioned studies have reported positive health benefits of HMOs during infancy, it is important to note that variations of HMO composition resulting from genetic polymorphisms, particularly fucosyltransferase genes (5), have been reported. Austin et al. (26) assessed 10 HMOs of HM samples obtained from Chinese women. They reported that with the exception of alpha-Tetrasaccharide (A-tetra), the majority of HMOs were above the limit of detection (LoD). Specifically, of the 446 samples evaluated, only 65 HM samples (∼14.6%) exhibited an A-tetra level greater than the LoD (A-tetra+). While the underlying biological mechanisms determining the levels of A-tetra in HM samples are largely unknown, Kobata (27) reported that A-tetra may only be present in mothers with a blood type A, which is consistent with the percentage of the Chinese population with an A blood type reported by Austin et al. (26). These findings raised 2 major considerations regarding the levels of A-tetra in HM. First, the presence (A-tetra+) or absence (A-tetra-) of A-tetra is associated with blood type, suggesting potential genetic origins. Second, given a large portion of HM samples are expected to be A-tetra-, statistical approaches accounting for this major factor should be considered. Taken together, in this study we aimed to examine the potential associations between HMOs and cognitive development, assessed by the Mullen Scales of Early Learning (MSEL; primary outcome) during infancy. Statistical approaches stratifying subjects based on the level of A-tetra were employed to determine whether associations of HMOs and early cognitive development differ between A-tetra+ and A-tetra- groups.

## Methods

Informed consent for both their own participation and the participation of their infant was obtained from parents prior to enrolling in the study. All study activities were approved by the University of North Carolina at Chapel Hill (UNC) and University of Minnesota (UMN) Institutional Review Boards. A subset of the Baby Connectome Project (BCP) who were younger than 3 years old were also enrolled in the BCP-Enriched study. Of subjects enrolled in BCP-Enriched study who were breastfed (including exclusive/predominant and mixed feeding) at the scheduled visits were included in the data analyses reported herein. Additional details of the BCP study can be found in Howell et al. (28) and **Supplemental Information 1**. The exclusively/predominantly breastfed infants were the infants who were fed less than 4 teaspoons or 20 g per day of nonformula and complementary foods/liquids (water, apple juice, etc.). In contrast, the mixed breastfed infants were the infants who were fed more than 50% human milk and some formula and other foods/liquids. The mixed formula–fed infants were those who were fed more than 50% formula, but also some human milk and other foods/liquids. Subjects were enrolled at both UNC (Site A) and UMN (Site B) using site-based research registries for identifying research participants between birth and 3 years of age. Recruitment strategies were further supplemented by local newborn nurseries, institutional centers with research activities focusing on early brain development, university listservs, and local flyers. The inclusion criteria were birth at gestational age 37–42 weeks, birth weight appropriate for gestational age, and absence of major pregnancy and delivery complications. The exclusion criteria included cases in which the child was adopted; the presence of a first-degree relative with autism, an intellectual disability, schizophrenia, or bipolar disorder; birth weight < 2000 grams; neonatal hypoxia (10-minute APGAR [Appearance, Pulse, Grimace, Activity, and Respiration] <5); illness requiring a newborn intensive care unit stay > 2 days; chromosomal or major congenital abnormality; abnormal magnetic resonance on a previous MRI; significant medical illness or developmental delay or significant medical and/or genetic conditions affecting growth, development, or cognition (including visual/hearing impairment); contraindication for MRI; and the mother having preeclampsia, having a placental abruption, living with HIV, or using alcohol or an illicit drug during pregnancy. Finally, both hearing and vision functions were also assessed.

### HM collection and macronutrient analyses

HM samples were obtained using a hospital-grade, electric Medela Symphony breast pump from the right breast at each visit. The milk samples were collected until no more HM could be extracted, so that the collective sample was representative of the nutrient concentration of the right breast for 1 feeding. In addition, collections were standardized to the second feed of the day whenever possible to avoid diurnal variation of milk nutrients. The weight and volume of milk collected were recorded, and the milk was then vortexed for 2 minutes at the highest speed. The volume of milk was measured using a graduated cylinder with special attention to avoid bubbles. Subsequently, 2.5 mL of milk was used for midinfrared spectroscopic analyses (MIRIS Human Milk Analyzer; Miris AB, Uppsala, Sweden). This step was to ensure that the total fat content (as an indicator of the quality of milk sampling) fell within the expected range. Finally, an aliquot of the minimum 30 mL of volume was transferred from the collection bottle to a 50 mL polypropylene Falcon tube. Using repeat pipette and an appropriate tip, 11 aliquots of 1 mL were made in 1-mL Eppendorf tubes and 9 aliquots of 2 mL were made in 2-mL Eppendorf tubes for storage in a −80°C freezer within 30 minutes from the end of the time of collection.

### HMO analyses

A representative 1-mL aliquot of collected HM was shipped on dry ice to Neotron Spa. (Italy) for HMO quantification by LC with fluorescence detection after labeling with 2-aminobenzamide using the method of Austin and Bénet (29). The following HMOs were quantified using standard curves with authentic HMO standards: 2’-FL, 3-fucosyllactose (3-FL), 3’-sialyllactose (3’-SL), 6’-SL, lacto-N-tetraose (LNT), lacto-N-neotetraose (LNNT), lacto-N-fucopentaose I (LNFP-I), and A-tetra.

The milk samples collected by Site A were all analyzed in 1 batch, whereas the milk samples collected by Site B were analyzed in 2 batches (1 along with the samples from Site A and the other with only samples from Site B).

### Mullen Scales of Early Learning

The MSEL, a widely used tool to assess infant cognitive development, is comprised of 5 subdomains, including fine motor, gross motor, visual reception, receptive language, and expressive language. An early learning composite (ELC) score corresponding to the Developmental Quotient score for infants can be derived using the scores of all subdomains, excluding the gross motor subdomain. The MSEL was administered at every visit by trained staff who were not blinded to subjects’ milk exposures.

### Statistical modeling and analyses

R version 3.6.3 (R Studio) was used for all statistical analyses. It is well known that HMO concentrations change across lactation (5). Thus, in order to remove the potential confounding effects from age, age effect was removed from the HMOs using smooth spline regression prior to conducting association analyses (30). Nevertheless, because the quantities of some of the HMOs are known to be below the LoD of the applied assays (undetectable), the age effect removal procedures were only applied to those with HMO concentrations greater than the LoD. In addition, log transformation was applied whenever needed in order to minimize the heteroskedasticity and satisfy the linear assumption of the linear models applied (31).

The associations between the 8 age-removed HMO concentrations and concurrently collected MSEL scores (as the response variable), including the ELC score and each of the subdomain MSEL *t*-scores, were tested. The data acquisition process and construction of the final data set for the association analysis are depicted in **Supplemental Figure 1**. To account for the longitudinal aspect, a random linear mixed effects model was used, with 2 random intercepts for the infant and MSEL examiner (32). As implicated by Verbyla (33), the restricted maximum likelihood (REML) approach can be used for model comparisons in addition to parameter estimation when the original likelihood is used with the REML estimates. Thus, the REML approach was used for fitting the models and for model comparison. The Akaike Information Criterion (AIC) was calculated using the original likelihood and the REML estimates (34). Potential HMO analysis batch and site effects were also controlled and were considered as binary variables.

Three statistical models were employed in our study, and detailed information is provided below. Model 1 was used to analyze all 8 of the HMOs for all samples without any stratifications.

The MSEL data represent the ELC or each of the subdomain scores. We applied Model 1 to assess potential associations between MSEL scores and all 8 measured HMOs for the entire population, without any stratifications.

As indicated above, our study aimed to determine whether associations between HMOs and cognitive development differ between A-tetra+ and A-tetra- groups. We stratified children as being either A-tetra+, meaning receiving HM containing more A-tetra than the LoD (4.4 mg/L), or otherwise as being A-tetra- (29). In addition, if A-tetra values were greater than 0 but lower than the LoD, these values were assigned as 0. For the A-tetra- subjects, the analysis batch and site were used as independent variables with 2 random intercepts, whereas in addition to the variables used for the A-tetra- subjects, 8 age-removed HMOs were also included for the A-tetra+ subjects.

Finally, to evaluate whether the HMOs exhibiting significant associations with MSEL have different associations at different ages, an indicator variable for age cutoff point was included as both main and interaction terms in Model 3. To choose the appropriate age cutoff point, the AIC was compared between models with different cutoff ages.

## Results

**FIGURE 1:**
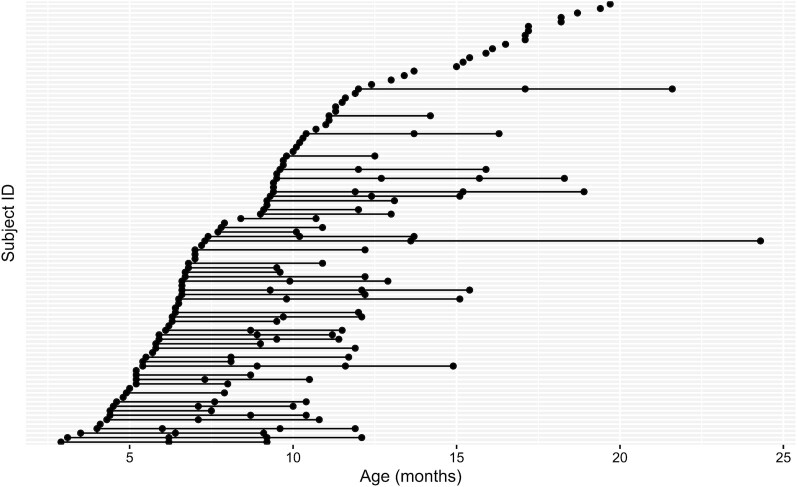
The ages of the 99 subjects when data were collected. Each filled circle indicates a study visit, and the horizontal lines represent subjects with multiple longitudinal visits.

A total of 99 mother-child dyads were enrolled in this study, and 183 HM samples were collected. The mean duration of breastfeeding was 14.4 ± 4.95 months. Among the 99 child participants, 80 participants were exclusively/predominantly breastfed, 15 were mixed breastfed, and 1 was mixed formula fed. Although feeding practice information was not available from the remaining 3 participants, they were all breastfed at the time of visits. With the exception of 1 participant who exhibited nystagmus, all children had normal vision and hearing tests. The age distributions for the study visits are shown in **Figure 1**. The numbers of participants with 1, 2, 3, and 4 study visits were 49, 24, 18, and 8, respectively. Detailed subject information; the weights, volumes, measured fat, carbohydrates, and energy of the milk samples; and 8 HMO concentrations for all, A-tetra+, and A-tetra- subjects are summarized in **Table 1**. HM samples from 2 subjects showed A-tetra levels greater than the LoD at some visits but 0s during the other visits (40, 28, 0, and 43 mg/L in 1 subject and 35, 0, 45, and 0 mg/L in the other subject). These 2 subjects were assigned to the A-tetra+ group. No significant differences in demographics were observed between the A-tetra+ and A-tetra- groups (*t*-test for all parameters, with the exception of household incomes, where a chi-square test of independence was used). The sex variable was not significant, and thus was not included in the model. While 3-FL concentrations were significantly different between the 2 groups, the inclusion or exclusion of 3-FL in the linear mixed effect models did not yield significant differences in the fitted results. Thus, results using all of the 8 HMOs are reported below. Relations of patterns of human milk exposure and HMO concentrations between A-tetra+ and A-tetra- groups were also studied (**Supplemental Tables 1** and **2**), and we concluded that no distinct difference existed between these groups (**Supplemental Information 2**). **Table 2** shows the MSEL ELC and individual subdomain scores, which are all within the anticipated normal ranges. While variables such as socioeconomic status and education levels of parents have been shown to impact early cognitive development of offspring, Tables 1 and 2 show that the demographic information and MSEL scores did not differ significantly between the A-tetra+ and A-tetra- subgroups, justifying the exclusion of these variables from the models.

**TABLE 1:** Demographic and human milk information of the participants^1^

		Total	A-tetra+	A-tetra-	*P* value^2^
Subjects		99 subjects	33 subjects	66 subjects	
Sex, male		41	19	22	
Age, months		10.13 (3.98)	10.85 (4.21)	9.8 (3.84)	0.11
Birth weights, kg		3.5 (0.42)	3.47 (0.48)	3.51 (0.39)	0.59
Birth lengths, cm		51.56 (2.39)	51.39 (2.18)	51.63 (2.47)	0.57
Gestational ages, months		9.27 (0.25)	9.2 (0.19)	9.32 (0.28)	0.21
% of vaginal births		0.88	0.8	0.93	
Human milk volume, mL		55.75 (31.94)	53.63 (30.05)	56.78 (32.89)	0.53
Human milk weight, g		56.29 (32.00)	52.97 (28.74)	57.87 (33.44)	0.33
Fat, g/100mL		5.02 (1.73)	5.03 (1.73)	5.02 (1.74)	0.97
Carbohydrate, g/100mL		6.87 (0.66)	6.77 (0.89)	6.92 (0.47)	0.27
Energy, kcal/100mL		78.21 (16.5)	77.57 (17.28)	78.59 (16.13)	0.74
Household income, USD, *n*	<25k	1	1	0	0.26
	25k–35k	4	0	4	
	35k–50k	2	2	0	
	50k–75k	17	5	12	
	75k–100k	14	3	11	
	100k–150k	19	7	12	
	150k–200k	12	4	8	
	>200k	9	5	4	
Mother education (grad level proportion)		0.61 (0.49)	0.64 (0.48)	0.59 (0.49)	0.59
HMO concentrations^3^	2’-FL	1276.72 (982.39)	1444.67 (717.31)	1200.75 (1075.16)	0.07
	3-FL	1580.37 (1031.32)	1340.37 (595.38)	1688.93 (1162.62)	0.01
	A-tetra	30.23 (75)	97.04 (108.07)	0 (0)	0
	3’-SL	190.42 (99.76)	202.22 (142.23)	185.08 (73.04)	0.39
	6’-SL	32.25 (47.4)	30.37 (39.78)	33.1 (50.59)	0.69
	LNT	401.08 (264.17)	397.6 (326.14)	402.65 (232.26)	0.92
	LNNT	64.94 (65.97)	70.35 (65.76)	62.5 (66.18)	0.46
	LNFP-I	248.42 (286.92)	256.08 (237.48)	244.96 (307.5)	0.79

1 Values show means (SDs). Abbreviations: A-tetra, alpha-Tetrasaccharide; A-tetra-, undetectable alpha-Tetrasaccharide; A-tetra+, alpha-Tetrasaccharide positive; LNFP-I, lacto-N-fucopentaose I; LNT, lacto-N-tetraose; LNNT, lacto-N-neotetraose; USD, United States dollar; 2’-FL, 2’-fucosyllactose; 3-FL, 3-fucosyllactose; 3’-SL, 3’-sialyllactose; 6’-SL, 6’-sialyllactose.

2 The *P* values are for the differences among subjects between A-tetra+ and A-tetra- groups using *t*-tests for all parameters, with the exception of household incomes, where the chi-square test of independence was used.

^3^Although our subjects had multiple visits, HMO concentration results shown in this table treated each sample independently.

**TABLE 2:** Mullen Scales of Early Learning scores of the participants^1^

	Total (*n* = 183)	A-tetra+ (*n* = 57)	A-tetra- (*n* = 126)	*P* value^2^
Composite score	106.18 (12.89)	104.39 (12.71)	106.99 (12.94)	0.2
Gross motor	51.58 (9.13)	53.53 (9.89)	50.7 (8.66)	0.07
Visual reception	53.6 (10)	52.37 (9.81)	54.16 (10.08)	0.26
Fine motor	54.66 (11.08)	53.65 (9.42)	55.11 (11.76)	0.37
Receptive language	50.52 (9.75)	50.49 (10.14)	50.54 (9.61)	0.98
Expressive language	53.42 (8.89)	52.05 (8.95)	54.04 (8.82)	0.17

1 Values are shown as means (SDs). Although our subjects had multiple Mullen Scales of Early Learning assessments over time, the results shown in this table treated each visit independently. Abbreviations: A-tetra-, undetectable alpha-Tetrasaccharide; A-tetra+, alpha-Tetrasaccharide positive.

2 The *P* values are for the differences among subjects between A-tetra+ and A-tetra- groups using *t*-tests.

**FIGURE 2:**
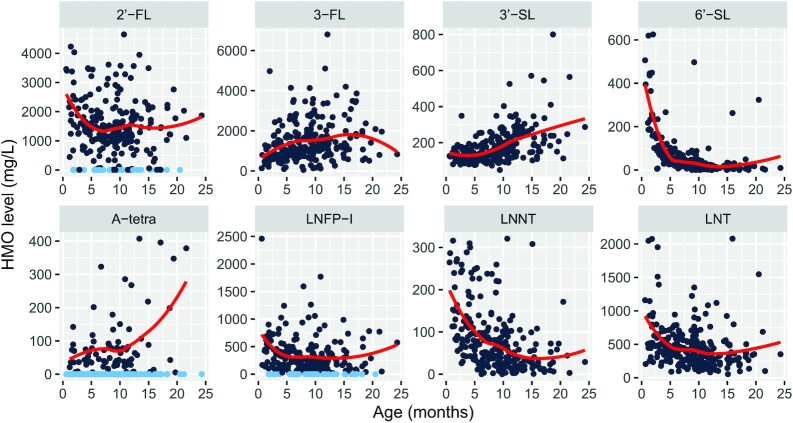
The distributions of the 8 HMOs in mg/L with age. Each HMO has 256 samples from 114 subjects from the final HMO data set. The light and dark blue filled circles in the 2’-FL, A-tetra, and LNFP-I scatter plots indicate human milk below and above the LoD, respectively. About 70% (129 samples), 21% (39 samples), and 12% (22 samples) of the human milk contained A-tetra, LNFP-I, and 2’-FL, respectively, below the LoDs of these HMOs (4.4, 2.0, and 3.9 mg/L for A-tetra, LNFP-I, and 2’-FL, respectively). The y-axis represents the HMO concentration in mg/L. The x-axis represents age in months. The red lines represent the mean values at different ages for each HMO, excluding the undetectable values for 2’-FL, A-tetra, and LNFP-I. The HMOs are highly age dependent, with 2’-FL, LNFP-I, LNNT, LNT, and 6’-SL decreasing and A-tetra, 3-FL, and 3’-SL increasing with age. For 2’-FL, 6’-SL, and LNFP-I, age has a significant quartic (adjusted *P* values < 0.001 for all 3) effect, whereas 3-FL, A-tetra, 3’-SL, LNT, and LNNT exhibit significant quadratic (adjusted *P* values < 0.01 for all 5) effects. Abbreviations: A-tetra, alpha-Tetrasaccharide; HMO, human milk oligosaccharide; LNFP-I, lacto-N-fucopentaose I; LoD, limit of detection; LNNT, lacto-N-neotetraose; LNT, lacto-N-tetraose; 2’-FL, 2’-Fucosyllactose; 3-FL, 3-fucosyllactose; 3’-SL, 3’-sialyllactose; 6’-SL, 6’-sialyllactose.

The HMO concentrations are highly age dependent (**Figure 2**), with 2’-FL, LNFP-I, LNNT, LNT, and 6’-SL decreasing with age, whereas A-tetra, 3-FL, and 3’-SL increase with age. For 2’-FL, 6’-SL, and LNFP-I, age has a significant quartic effect [adjusted *P* values < 0.001 for all 3 effect sizes (EFs): 1837.19, 338.66, and 996.60 for quartic age, respectively; 95% CIs, 974.16–2701.50, 217.20–459.51, and 578.77–1412.53 for quartic age, respectively), whereas 3-FL, A-tetra, 3’-SL, LNT, and LNNT exhibited significant quadratic effects (adjusted *P* values < 0.01 for all 5 EFs, -1655.18, 56.27, 170.88, 1232.48, and 200.51, respectively; 95% CIs, -2729.39 to -582.43, 21.81–90.82, 51.72–288.45, 697.81–1773.43, and 103.11–299.45, respectively). Notably, about 70% (129 samples), 21% (39 samples), and 12% (22 samples) of the HM contained A-tetra, LNFP-I, and 2’-FL below the LoDs (4.4, 2.0, and 3.9 mg/L, respectively) (29). Among these 3 HMOs, HM samples exhibiting 2’-FL levels below the LoD also had undetectable LNFP-I, and all the undetectable LNFP-I samples also had undetectable A-tetra.

For age-removed 3’-SL, log transformation was necessary in order to minimize the heteroskedasticity and satisfy the linearity assumption. Comparisons between distributions with and without the log-transformation of 3’-SL are provided in **Supplemental Information 3**. Furthermore, evaluations of intra-subject variability of 3’-SL from before and after age effects were removed are provided in Supplemental Information 3.

No significant associations among cognition and HMO concentrations were observed when all samples without stratifications were analyzed using Model 1. In contrast, a significantly positive association existed between the ELC scores and age-removed 3’-SL (*P* = 0.002; EF, 13.12; 95% CI, 5.36–20.80), with a power of 0.89 when children were stratified into A-tetra+ and A-tetra- groups (Model 2), suggesting that a higher 3’-SL concentration is associated with a higher ELC score in children receiving HM with measurable levels of A-tetra (**Table 3**). More specifically, controlling for other HMOs and site and batch effects, the ELC score increased by 13.12 for every unit increase of the age-removed 3’-SL. We further determined whether the observed positive association between 3’-SL and the MSEL ELC score was specific to particular cognitive subdomains by evaluating each MSEL subdomain score using Model 2. The Holm-Bonferroni method was used to correct for multiple comparisons (35). Significant and positive associations between 3’-SL and the receptive (adjusted *P* = 0.015; EF, 9.95; 95% CI, 3.91–15.99) and expressive (adjusted *P* = 0.048; EF, 7.53; 95% CI, 2.51–13.79) language subscores were observed, with powers of 0.86 and 0.73, respectively, (Table 3). In other words, with other variables controlled, a unit increase in the age-removed 3’-SL was associated with 9.95 and 7.53 higher receptive and expressive language *t*-scores, respectively. In addition to applying Model 2, we also separately analyzed the A-tetra+ and A-tetra- subjects using Model 1. Similar results as those observed using Model 2 were found. Nevertheless, a single model (Model 2) comprising all data had more statistical power and was more robust than Model 1 when separately analyzing the A-tetra+ and A-tetra- groups. More detailed discussion is provided in **Supplemental Information 4**.

**TABLE 3:** Statistical results of associations between ELC, expressive language, and receptive language scores and HMOs^1^

		Intercept	Batch 1 Site B	Batch 1 Site A	2’-FL	3-FL	3’-SL	6’-SL	LNT	LNNT	LNFP-I	A-tetra+	A-tetra-
ELC score (*n* = 183)	Estimate	108.213	−9.38	−3.316	−0.001	0.005	13.119	0.038	−0.012	0.048	0.008	−0.022	2.386
	SE	2.628	2.358	2.505	0.005	0.004	4.087	0.08	0.014	0.035	0.019	0.02	2.63
	*t*	41.172	−3.978	−1.324	−0.246	1.25	3.21	0.475	−0.854	1.381	0.417	−1.111	0.907
	*P* value	<0.001^3^	<0.001^3^	0.189	0.806	0.213	0.002^3^	0.635	0.395	0.169	0.677	0.269	0.366
Receptive language (*n* = 183)	Estimate	51.287	−3.953	−0.47	−0.004	0	9.952	0.022	−0.011	0.017	0.008	−0.008	0.679
	SE	2.076	1.863	1.978	0.004	0.003	3.228	0.063	0.011	0.028	0.015	0.015	2.077
	*t*	24.708	−2.122	−0.238	−0.928	0.015	3.083	0.358	−1.019	0.602	0.531	−0.498	0.327
	Adjusted *P* value^2^	<0.001^3^	0.108	1	1	1	0.015^3^	1	1	1	1	1	1
Expressive language (*n* = 183)	Estimate	53.684	−4.091	−0.258	−0.001	0.003	7.534	0.053	−0.01	0.009	0.008	0.002	2.048
	SE	2.241	1.972	2.575	0.004	0.003	2.946	0.057	0.01	0.025	0.013	0.014	1.887
	*t*	23.96	−2.074	−0.1	−0.302	0.869	2.558	0.94	−1.057	0.371	0.595	0.144	1.085
	Adjusted *P* value^2^	<0.001^3^	0.108	1	1	1	0.048^3^	1	1	1	1	1	1

1 Data show fitted results using a random linear mixed effects model with 2 random intercepts. Abbreviations: A-tetra-, undetectable alpha-Tetrasaccharide; A-tetra+, alpha-Tetrasaccharide positive; ELC, early learning composite; HMO, human milk oligosaccharide; LNFP-I, lacto-N-fucopentaose I; LNT, lacto-N-tetraose; LNNT, lacto-N-neotetraose; 2’-FL, 2’-fucosyllactose; 3-FL, 3-fucosyllactose; 3’-SL, 3’-sialyllactose; 6’-SL, 6’-sialyllactose.

2 The adjusted *P* value for receptive language and expressive language are calculated using the Holm-Bonferroni method.

3 Statistically significant at *P* < 0.05.

For the batch and site effects, subjects from batch 1 of Site B had a 9.38 significantly lower (*P* < 0.001; 95% CI, -13.90 to -4.96) ELC score when compared to those from batch 2 of Site B (Table 3). The gross motor and visual reception *t*-scores were 6.80 and 9.06 lower, respectively, in Site B subjects from batch 1 than in those from batch 2 (both were significant differences at adjusted *P* values < 0.001; 95% CI, -9.87 to -3.61 and -12.51 to -5.67, respectively). The visual reception *t*-score was 5.85 lower in Site A subjects compared to the Site B subjects from batch 2 (significant at an adjusted *P* value of 0.02; 95% CI, -9.55 to -2.23). The results of the gross motor and visual reception *t*-scores are not included in Table 3.

**FIGURE 3:**
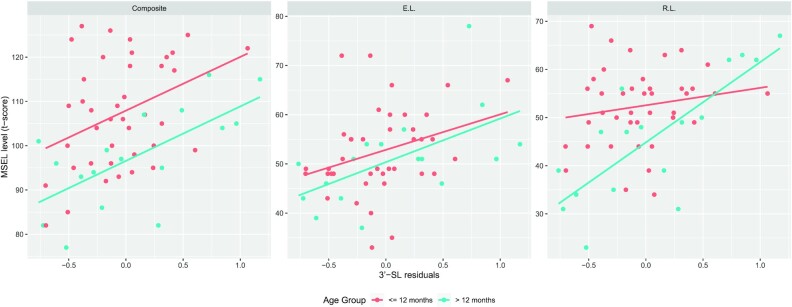
Relationship between 3’-SL and composite score, E.L., and R.L. by age groups ≤12 months (red) and >12 months (blue), respectively] among the A-tetra+ group. Each point represents the samples from A-tetra+ subjects. There are 40 samples in the younger age group and 17 in the older group. The red and blue lines show the fitted linear regression lines using linear mixed effects models by age groups. The associations of age-removed 3’-SL on the composite score were consistent throughout ages, but younger subjects had higher composite scores for the same age-removed 3’-SL compared to the older subjects. For the R.L. *t*-scores, the increments of the *t*-scores per age-removed 3’-SL were significantly greater for the older subjects. Specifically, for 1 increase in the age-removed 3’-SL, the R.L. *t*-score increased by about 1.82 and 16.74 for infants younger and older than 1 year old, respectively, resulting in the difference of 14.93 between the younger and the older groups. The adjusted *P* value for the interaction term between age and age-removed 3’-SL was 0.03 (95% CI, -25.29 to -4.24). The *P* value for the interaction term for composite score was 0.538, and the adjusted *P* value for the interaction term for E.L. was 1. Thus, for the composite score and E.L., the effect of age-removed 3’-SL was consistent between age groups. The y-axis represents the MSEL *t*-score levels and the x-axis represents the residuals of 3’-SL. Abbreviations: A-tetra+, detectable alpha-Tetrasaccharide; E.L., expressive language; MSEL, Mullen Scales of Early Learning; R.L., receptive language; 3’-SL, 3’-sialyllactose.

We further studied whether the observed positive associations of 3’-SL and the language subdomain scores had interactions with age. Using the AIC together with attempts to balance the sample sizes of the 2 age subgroups, 12 months was chosen as the cutoff point. **Figure 3** shows that among the A-tetra+ subjects, the associations of age-removed 3’-SL levels on the ELC scores were consistent across ages, but younger subjects had higher ELC scores for the same age-removed 3’-SL level compared to the older subjects. For the receptive language *t*-scores, the increments of the *t*-scores per age-removed 3’-SL level were significantly greater for the older subjects. Specifically, for 1 increase in the age-removed 3’-SL level, the receptive language *t*-score increased by about 1.82 and 16.74 for infants younger and older than 1 year old, respectively, resulting in a difference of 14.93 between the younger and the older groups (adjusted *P* = 0.03 for the interaction between age and age-removed 3’-SL; 95% CI, -25.29 to -4.24).

## Discussion

The present study evaluated the potential associations between 8 HMOs and cognitive development during infancy. Based on the A-tetra level in HM that they received, children were stratified into A-tetra+ and A-tetra- subgroups. Levels of 3’-SL were significantly and positively associated with the ELC scores in A-tetra+ subjects, and the associations were specific to expressive and receptive language scores. Furthermore, larger effects of 3’-SL on receptive language were observed for children older than 12 months as compared to for those younger than 12 months. To the best of our knowledge, these are the first reported human results demonstrating positive associations between 3’-SL and cognitive development, particularly language development, during infancy.

### Development of language ability during infancy

Because language is a trait only humans possess, it is not surprising that all previously reported preclinical studies were unable to uncover the potential benefits of HMOs on language function. The MSEL assesses 2 specific language functions: receptive and expressive language. Developmentally, it has been widely recognized that cognitive maturation follows different temporal orders depending on the functional domains (36–38). Therefore, we investigated a plausible age-related association between 3’-SL and language function. We found that the associations between expressive language and 3’-SL were statistically indistinguishable between infants younger and older than 12 months of age. In contrast, larger effects of 3’-SL on receptive language were observed for children older than 12 months. These findings may not be surprising given the developmental characteristics of language functions. Specifically, although primitive receptive language functions (vocal learning) emerge during the first year of life, auditory comprehension, memory, and sequencing are required for more matured receptive language ability. Auditory function undergoes rapid maturation in the first year of life (36), whereas working memory has been shown to emerge by year 1 (39), supporting our findings that the association between 3’-SL and receptive language could be greater beyond 1 year of age. In contrast, expressive language follows a more protracted developmental trajectory (40, 41). The mean age of our cohort was about 10 months old, potentially leading to insufficient age coverage to uncover the age interaction between 3’-SL and expressive language.

### Secretor and Lewis statuses

Differences in HMO secretion in mothers based on their Lewis blood group have been reported (42, 43). Among the blood groups, secretor-positive (Se+) and Lewis positive (Le+), mothers secreted all of the oligosaccharides examined, but secretor-negative (Se-), Lewis-negative (Le-), and Se+/Le- mothers did not secrete α1,2-fucosylated compounds and α1,4-fucosyloligosaccharides. Newburg et al. (44) further demonstrated that the differences in blood groups resulted in different compositions of HM and affected the immune system. To further determine whether the associations between HMOs and early cognitive development depend on the secretor and Lewis statuses, exploratory analyses were conducted. Results and discussion are provided in Supplemental Information 4. In short, given the limited sample sizes of the Se+/Le- and Se-/Le- groups (**Supplemental Table 3)**, analyses were focused on the Se+/Le+ and Se-/Le+ groups. No significant association was observed for Se-/Le+ group, whereas significant associations between 3’-SL and the ELC score (*P* = 0.02) and receptive language subscore (adjusted *P* = 0.045) were observed for the Se+/Le+ group using Model 1. We further evaluated whether these findings were independent of the A-tetra status using Model 2 for the Se+/Le+ group. Significant associations of 3’-SL with ELC (*P* = 0.001), receptive language (adjusted *P* = 0.015), and expressive language (adjusted *P* = 0.016) scores were observed in the A-tetra+ group. Therefore, the positive associations observed using Model 1 are most likely driven by the A-tetra+ subjects. Together, it appears that secretor and Lewis statuses do not account for A-tetra levels in our study population. Nevertheless, studies with larger and balanced sample sizes among the 4 Lewis blood groups are warranted to better understand the implications of genetic polymorphisms and early cognitive development.

### A-tetra+ and A-tetra-

Austin et al. (26) reported that ∼16.7% of HM samples obtained from Chinese women were A-tetra+, and these women were all blood type A. In the context of our study, the percentage of the US population with blood type A ranges between ∼34% and 36% (45), which is consistent with our results, where 33.3% of mothers had A-tetra+ HM, suggesting that the A-tetra level may be a proxy for blood type, and consequently HM grouping. However, it is worth noting that 2 women in our study showed temporally varied A-tetra levels in the HM samples between visits. Therefore, other biological underpinnings leading to women secreting detectable compared with undetectable A-tetra should be considered. We offer hypothetical explanations. Glycosylation disorder, a defection of glucosidase I, was shown to be present in patients with accumulated A-tetra in their urine (46). Furthermore, soluble Klotho (sKlotho), which is an ectodomain shedding of antiaging membrane, was shown to affect the α2–3-sialyllactose in glycolipid gangliosides and possess cellular effects interfered by lipid rafts, where the α2–3-sialyllactose are supplemented (47). Thus, it is conceivable that both glucosidases and sKlotho play a role between women secreting detectable compared with undetectable A-tetra. Nevertheless, future studies will be needed to gain additional insights into how A-tetra concentrations may vary with time.

### Beyond 3’-SL

Berger et al. (24) reported that higher exposures of 2’-FL relative to other HMOs at 1 month of age were associated with better cognitive ability, assessed at 24 months of age. Oliveros et al. (25) showed a positive association between 2’-FL levels at 1 month of age and 6-month motor scores, whereas 6’-SL levels were positively associated with cognitive and motor scores at 18 months. However, no association was found between 2’-FL nor 6’-SL and cognitive development in our study. Although many factors may explain the discrepancies between our results and those reported by these 2 studies, the differences in the study designs may account for the observed discrepancies. Specifically, both the Berger et al. (24) and Oliveros et al. (25) studies utilized a classic longitudinal design, whereas an accelerated longitudinal design was employed in our study. As a result, data from our cohort cannot be used to evaluate how differences in exposure to HMOs at early ages may lead to diverse cognitive outcomes at later ages. Instead, association analyses of 8 commonly reported HMOs and cognitive development were conducted in our study.

While we reported the results of 8 quantitative HMOs, HMOs with the 24 Maltotriose quantitative HMOs were also analyzed. Given the limited sample size in our study, exploratory analyses were conducted using variable selection via backward elimination. The main findings were similar to those reported above (**Supplemental Information 5**).

### Limitations

Our study has several potential limitations. We did not collect data on either the time taken for the acquisition of HM samples after the last feeding nor the frequency of breastfeeding. Factors other than socioeconomic status and maternal factors, such as the first language spoken at home, multiple birth status, and maternal parity, might modulate children's cognitive ability. Lack of diversity in the study population hampers the generalizability of our results. The use of an accelerated longitudinal design makes it difficult for prediction analyses. Insufficient age coverage hinders the discovery of any age interaction between 3’-SL and expressive language. The biological links between 3’-SL and language development remain elusive. Finally, since the study focuses on the role of oligosaccharides, our data cannot determine whether other milk components, such as fatty acids, also influence cognitive development. Detailed discussions of these limitations are provided in **Supplemental Information 6**.

### Conclusion

When children were stratified based on A-tetra levels in the HM that they received, a significant positive association between 3’-SL and the ELC score was observed in A-tetra+ subjects. This association was driven by the receptive and expressive language subdomain scores. Our results support the potential cognitive benefits of HMOs in the A-tetra+ subgroups of infants.

## Supplementary Material

nqab103_Supplemental_File

## Data Availability

Data described in the manuscript, code book, and analytic code will be made available upon request pending application and approval by the authors.

## Notes

This study was supported in part by NIH grants (U01MH110274 to WL and JTE; R01MH104324 and MH1044324-03S1 to JTE; MH015755 to BRH's trainee) and a grant from Nestlé Product Technology Center-Nutrition, Société des Produits Nestlé SA, Switzerland (to WL). As indicated in the manuscript, the Baby Connectome Project (BCP) is an NIH-funded study. Therefore, all of the experimental designs, including inclusion and exclusion criteria, the sampling strategies, subject grouping strategies, recruitment/retention approaches, and the age-appropriate cognitive assessment instruments and parent-report questionnaires used were formulated and implemented by the investigators of BCP, independent of the sponsors (Nestle) of the BCP-Enriched study. In contrast, the experimental designs for collecting additional biosamples, including human milk and the analysis approaches of the collected human milk, were done in collaboration with scientists from the sponsors. The statistical analyses conducted in this manuscript were independent of sponsors. Nevertheless, the sponsors were involved in data interpretation and thus included as authors (JH and NS).

Supplemental Information 1–6, Supplemental Figure 1, and Supplemental Tables 1–3 are available from the “Supplementary data” link in the online posting of the article and from the same link in the online table of contents at https://academic.oup.com/ajcn/.

Abbreviations used:A-tetra, alpha-Tetrasaccharide; A-tetra-, undetectable alpha-Tetrasaccharide; A-tetra+, alpha-Tetrasaccharide positive; AIC, Akaike Information Criterion; BCP, Baby Connectome Project; EF, effect size; ELC, early learning composite; Fuc, fucose; HM, human milk; HMO, human milk oligosaccharide; Le-, Lewis negative; Le+, Lewis positive; LNFP-I, lacto-N-fucopentaose I; LNNT, lacto-N-neotetraose; LNT, lacto-N-tetraose; LoD, limit of detection; MSEL, Mullen Scales of Early Learning; REML, restricted maximum likelihood; Se-, secretor negative; Se+, secretor positive; sKlotho, soluble Klotho; SL, sialyllactose; UMN, University of Minnesota; UNC, University of North Carolina at Chapel Hill; 2’-FL, 2’-fucosyllactose; 3-FL, 3-fucosyllactose; 3’-SL, 3’-sialyllactose; 6’-SL, 6’-sialyllactose.

## References

[1] Victora CG , Bahl R, Barros AJD, França GVA, Horton S, Krasevec J, Murch S, Sankar MJ, Walker N, Rollins NC. Breastfeeding in the 21st century: Epidemiology, mechanisms, and lifelong effect. Lancet North Am Ed. 2016;387:475–90. 10.1016/S0140-6736(15)01024-7 26869575

[2] Cheng L , Akkerman R, Kong C, Walvoort MTC, de Vos P. More than sugar in the milk: Human milk oligosaccharides as essential bioactive molecules in breast milk and current insight in beneficial effects. Crit Rev Food Sci Nutr. 2021:61(7);1184–1200. 32329623 10.1080/10408398.2020.1754756

[3] Walsh C , Lane JA, van Sinderen D, Hickey RM. Human milk oligosaccharides: Shaping the infant gut microbiota and supporting health. J Funct Foods. 2020;72:104071. 10.1016/j.jff.2020.104074 PMC7332462 32834834

[4] Walsh C , Lane JA, van Sinderen D, Hickey RM. From lab bench to formulated ingredient: Characterization, production, and commercialization of human milk oligosaccharides. J Funct Foods. 2020;72:104052. 10.1016/j.jff.2020.104074 PMC7332462 32834834

[5] Samuel TM , Binia A, de Castro CA, Thakkar SK, Billeaud C, Agosti M, Al-Jashi I, Costeira MJ, Marchini G, Martínez-Costa C et al. Impact of maternal characteristics on human milk oligosaccharide composition over the first 4 months of lactation in a cohort of healthy European mothers. Sci Rep. 2019;9:11767. 31409852 10.1038/s41598-019-48337-4 PMC6692355

[6] Sela DA . Bifidobacterial utilization of human milk oligosaccharides. Int J Food Microbiol. 2011;149:58–64. 21342711 10.1016/j.ijfoodmicro.2011.01.025

[7] Matsuki T , Yahagi K, Mori H, Matsumoto H, Hara T, Tajima S, Ogawa E, Kodama H, Yamamoto K, Yamada T et al. A key genetic factor for fucosyllactose utilization affects infant gut microbiota development. Nat Commun. 2016;7:11939. 27340092 10.1038/ncomms11939 PMC4931012

[8] Berger B , Porta N, Foata F, Grathwohl D, Delley M, Moine D, Charpagne A, Siegwald L, Descombes P, Alliet P et al. Linking human milk oligosaccharides, infant fecal community types, and later risk to require antibiotics. mBio. 2020;11:e03196–19. 32184252 10.1128/mBio.03196-19 PMC7078481

[9] Ross SA , Lane JA, Marotta M, Kavanaugh D, Ryan JT, Joshi L, Hickey RM. The role of oligosaccharides in host-microbial interactions for human health. J Clin Gastroenterol. 2016;50(Suppl 2):S131–2. 27741156 10.1097/MCG.0000000000000694

[10] Urashima T , Hirabayashi J, Sato S, Kobata A. Human milk oligosaccharides as essential tools for basic and application studies on galectins. Trends Glycosci Glycotechnol. 2018;30:SE51–65.

[11] Kulinich A , Liu L. Human milk oligosaccharides: The role in the fine-tuning of innate immune responses. Carbohydr Res. 2016;432:62–70. 27448325 10.1016/j.carres.2016.07.009

[12] Triantis V , Bode L, van Neerven RJJ. Immunological effects of human milk oligosaccharides. Front Pediatr. 2018;6:190. 30013961 10.3389/fped.2018.00190 PMC6036705

[13] Puccio G , Alliet P, Cajozzo C, Janssens E, Corsello G, Sprenger N, Wernimont S, Egli D, Gosoniu L, Steenhout P. Effects of infant formula with human milk oligosaccharides on growth and morbidity: A randomized multicenter trial. J Pediatr Gastroenterol Nutr. 2017;64:624–31. 28107288 10.1097/MPG.0000000000001520 PMC5378003

[14] Bode L . Human milk oligosaccharides: Every baby needs a sugar mama. Glycobiology. 2012;22:1147–62. 22513036 10.1093/glycob/cws074 PMC3406618

[15] Sakai F , Ikeuchi Y, Urashima T, Fujihara M, Ohtsuki K, Yanahira S. Effects of feeding sialyllactose and galactosylated N-acetylneuraminic acid on swimming learning ability and brain lipid composition in adult rats. J Appl Glycosci. 2006;53:249–54.

[16] Tarr AJ , Galley JD, Fisher SE, Chichlowski M, Berg BM, Bailey MT. The prebiotics 3′sialyllactose and 6′sialyllactose diminish stressor-induced anxiety-like behavior and colonic microbiota alterations: Evidence for effects on the gut-brain axis. Brain Behav Immun. 2015;50:166–77. 26144888 10.1016/j.bbi.2015.06.025 PMC4631662

[17] Oliveros E , Vázquez E, Barranco A, Ramírez M, Gruart A, Delgado-García JM, Buck R, Rueda R, Martín MJ. Sialic acid and sialylated oligosaccharide supplementation during lactation improves learning and memory in rats. Nutrients. 2018;10:1519. 10.3390/nu10101519 PMC6212975 30332832

[18] Jacobi SK , Yatsunenko T, Li D, Dasgupta S, Yu RK, Berg BM, Chichlowski M, Odle J. Dietary isomers of sialyllactose increase ganglioside sialic acid concentrations in the corpus callosum and cerebellum and modulate the colonic microbiota of formula-fed piglets. J Nutr. 2016;146:200–8. 26701794 10.3945/jn.115.220152

[19] Mudd AT , Fleming SA, Labhart B, Chichlowski M, Berg BM, Donovan SM, Dilger RN. Dietary sialyllactose influences sialic acid concentrations in the prefrontal cortex and magnetic resonance imaging measures in corpus callosum of young pigs. Nutrients. 2017;9:1219. 10.3390/nu9121297 PMC5748748 29182578

[20] Obelitz-Ryom K , Bering SB, Overgaard SH, Eskildsen SF, Ringgaard S, Olesen JL, Skovgaard K, Pankratova S, Wang B, Brunse A et al. Bovine milk oligosaccharides with sialyllactose improves cognition in preterm pigs. Nutrients. 2019;11:1335. 10.3390/nu11061335 PMC6628371 31207876

[21] Vazquez E , Barranco A, Ramirez M, Gruart A, Delgado-Garcia JM, Jimenez ML, Buck R, Rueda R. Dietary 2’-fucosyllactose enhances operant conditioning and long-term potentiation via gut-brain communication through the vagus nerve in rodents. PLOS One. 2016;11:e0166070. 27851789 10.1371/journal.pone.0166070 PMC5113009

[22] Oliveros E , Ramirez M, Vazquez E, Barranco A, Gruart A, Delgado-Garcia JM, Buck R, Rueda R, Martin MJ. Oral supplementation of 2′-fucosyllactose during lactation improves memory and learning in rats. J Nutr Biochem. 2016;31:20–7. 27133420 10.1016/j.jnutbio.2015.12.014

[23] Vázquez E , Barranco A, Ramírez M, Gruart A, Delgado-García JM, Martínez-Lara E, Blanco S, Martín MJ, Castanys E, Buck R et al. Effects of a human milk oligosaccharide, 2′-fucosyllactose, on hippocampal long-term potentiation and learning capabilities in rodents. J Nutr Biochem. 2015;26:455–65. 25662731 10.1016/j.jnutbio.2014.11.016

[24] Berger PK , Plows JF, Jones RB, Alderete TL, Yonemitsu C, Poulsen M, Ryoo JH, Peterson BS, Bode L, Goran MI. Human milk oligosaccharide 2’-fucosyllactose links feedings at 1 month to cognitive development at 24 months in infants of normal and overweight mothers. PLOS One. 2020;15:e0228323. 32049968 10.1371/journal.pone.0228323 PMC7015316

[25] Oliveros E , Martín M, Torres-Espínola F, Segura-Moreno M, Ramírez M. Human milk levels of 2-fucosyllactose and 6-sialyllactose are positively associated with infant neurodevelopment and are not impacted by maternal BMI or diabetic status. J Nutr Food Sci. 2021;4:100024.

[26] Austin S , De Castro CA, Bénet T, Hou Y, Sun H, Thakkar SK, Vinyes-Pares G, Zhang Y, Wang P. Temporal change of the content of 10 oligosaccharides in the milk of Chinese urban mothers. Nutrients. 2016;8:346. 10.3390/nu8060346 PMC4924187 27338459

[27] Kobata A . Structures and application of oligosaccharides in human milk. Proc Jpn Acad Ser B Phys Biol Sci. 2010;86:731–47. 10.2183/pjab.86.731 PMC3066539 20689231

[28] Howell BR , Styner MA, Gao W, Yap P-T, Wang L, Baluyot K, Yacoub E, Chen G, Potts T, Salzwedel A et al. The UNC/UMN Baby Connectome Project (BCP): An overview of the study design and protocol development. Neuroimage. 2019;185:891–905. 29578031 10.1016/j.neuroimage.2018.03.049 PMC6151176

[29] Austin S , Bénet T. Quantitative determination of non-lactose milk oligosaccharides. Anal Chim Acta. 2018;1010:86–96. 29447675 10.1016/j.aca.2017.12.036

[30] Hastie TJ , Tibshirani RJ. Generalized additive models, Boca Raton, Florida, USA. CRC Press; 1990.

[31] Bartlett MS . The use of transformations. Biometrics. 1947;3:39–52. 20240416

[32] Laird NM , Ware JH. Random-effects models for longitudinal data. Biometrics. 1982;38:963–74. 7168798

[33] Verbyla AP . A note on model selection using information criteria for general linear models estimated using REML. Aust NZ J Stat. 2019;61:39–50.

[34] Akaike H . A new look at the statistical model identification. IEEE T Automat Contr. 1974;19:716–23.

[35] Miller RGJ . Simultaneous statistical inference. New York: Springer; 2012.

[36] McGurk H , MacDonald J. Auditory-visual coordination in the first year of life. Int J Behav Dev. 1978;1:229–39.

[37] Toga AW , Thompson PM, Sowell ER. Mapping brain maturation. Trends Neurosci. 2006;29:148–59. 16472876 10.1016/j.tins.2006.01.007 PMC3113697

[38] Thompson RA , Nelson CA. Developmental science and the media: Early brain development. Am Psychol. 2001;56:5–15. 11242988 10.1037/0003-066x.56.1.5

[39] Kibbe MM , Leslie AM. What's the object of object working memory in infancy? Unraveling “what” and “how many.” Cognit Psychol. 2013;66:380–404. 23770623 10.1016/j.cogpsych.2013.05.001

[40] Gard A , Gilman L, Gorman J. Speech and language development chart. (2nd ed), Pro-Ed, Austin, TX , USA. 1993.

[41] Owens RE . Language development : An introduction. Harlow, UK: Pearson Education; 2016.

[42] Thurl S , Munzert M, Henker J, Boehm G, Müller-Werner B, Jelinek J, Stahl B. Variation of human milk oligosaccharides in relation to milk groups and lactational periods. Br J Nutr. 2010;104:1261–71. 20522272 10.1017/S0007114510002072

[43] Thurl S , Henker J, Siegel M, Tovar K, Sawatzki G. Detection of four human milk groups with respect to Lewis blood group dependent oligosaccharides. Glycoconj J. 1997;14:795–9. 9511984 10.1023/a:1018529703106

[44] Newburg DS , Ruiz-Palacios GM, Altaye M, Chaturvedi P, Meinzen-Derr J, Guerrero M de L, Morrow AL. Innate protection conferred by fucosylated oligosaccharides of human milk against diarrhea in breastfed infants. Glycobiology. 2004;14:253–63. 14638628 10.1093/glycob/cwh020

[45] Garratty G , Glynn SA, McEntire R; Retrovirus Epidemiology Donor Study. ABO and Rh(D) phenotype frequencies of different racial/ethnic groups in the United States. Transfusion. 2004;44:703–6. 15104651 10.1111/j.1537-2995.2004.03338.x

[46] De Praeter CM , Gerwig GJ, Bause E, Nuytinck LK, Vliegenthart JFG, Breuer W, Kamerling JP, Espeel MF, Martin J-JR, De Paepe AM et al. A novel disorder caused by defective biosynthesis of N-linked oligosaccharides due to glucosidase I deficiency. Am J Hum Genet. 2000;66:1744–56. 10788335 10.1086/302948 PMC1378052

[47] Wright JD , An S-W, Xie J, Yoon J, Nischan N, Kohler JJ, Oliver N, Lim C, Huang C-L. Modeled structural basis for the recognition of α2-3-sialyllactose by soluble Klotho. FASEB J. 2017;31:3574–86. 28442546 10.1096/fj.201700043R PMC5503716

