# Supplementary material for: Human milk 3’-Sialyllactose is positively associated with language development during infancy
Source: Am J Clin Nutr. 2021 May 21;114(2):588–97. doi: 10.1093/ajcn/nqab103 (PMC8326052; doi:10.1093/ajcn/nqab103)
Supplement: nqab103_Supplemental_File [file nqab103_supplemental_file.docx]

## Human milk 3’-Sialyllactose positively associates with language development during infancy

## Cho et al.

## Online Supplementary Material

**Supplementary Figure 1: Study flowchart**

**
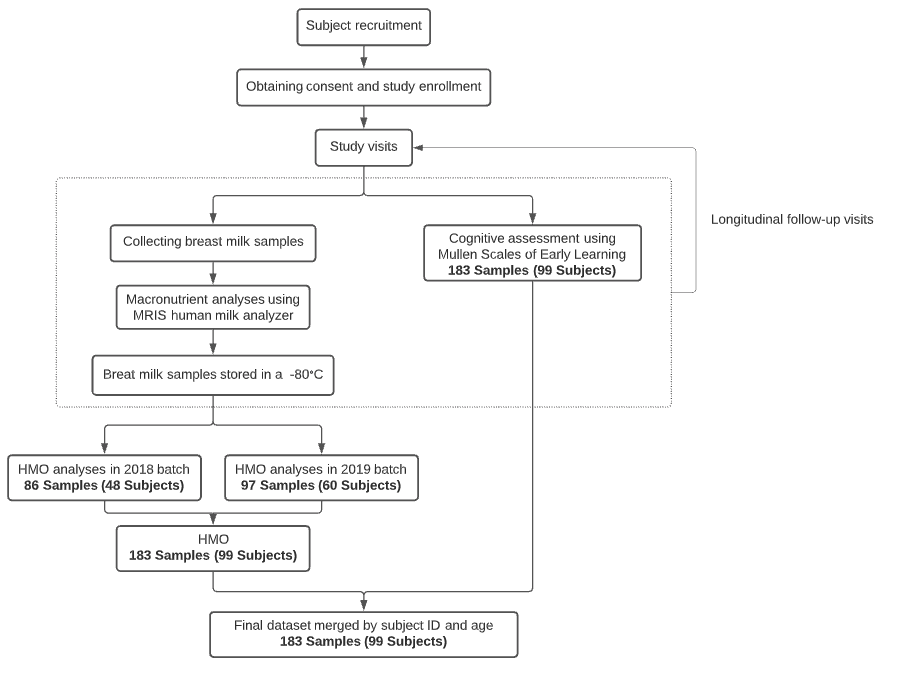
**

**Supplementary Information-1: Baby Connectome Project**

The Baby Connectome Project (BCP) is an NIH funded study (U01MH110274 (Lin, Elison)) focusing on characterizing early brain development in typically developing children. Detailed descriptions of the study goals, study designs and protocols are provided in Howell et al. (1). In short, BCP aims to collect high quality MR images, including structural, functional and diffusion tenor images and a comprehensive battery of age-appropriate behavioral assessments and parent report questionnaires from typically developing children between 0 and 5 years of age. An accelerated longitudinal design was employed where the ages of the first visit, time intervals between two adjacent visits, and the duration in the study varied among participants depending on the group assignment of a given subject. A subset of the BCP subjects who were younger than 3 years old were also enrolled in the BCP-Enriched study. A total of 332 participants were enrolled in the BCP-Enriched study and 62 withdrew. Attrition (18.6%) was largely due to the inability for the family to find time for the study, or their move away from the study area, which was unrelated to the nutritional aspects of the study. Nevertheless, it should be noted that we cannot rule out factors other than the aforementioned reasons that may also contribute to the attrition.

**Supplementary Information-2: Relations of patterns of human milk exposure and HMO concentrations between A-tetra+ and A-tetra- groups**

We further considered if concentrations of HMOs differ within and between different feeding practices for A-tetra+ and A-tetra- groups, respectively. We first compared age effects removed HMO concentrations between A-tetra+ and A-tetra- groups within the same feeding practice, exclusive/predominant and mixed breastfeeding, respectively **(Supplementary Table 1)**. Subsequently, the comparisons between the two breastfeeding practices within either A-tetra+ or A-tetra- group were conducted (**Supplementary Table 2**). T-test was used and p-values are provided. Mixed formula fed infant was not considered here because there was only one subject who was in the A-tetra+ group. It appears that the patterns of human milk exposure might be associated with HMO concentrations between A-tetra+ and A-tetra- groups. Statistically significant differences were observed for age-removed 3-FL and LNNT between A-tetra+ and A-tetra- groups within exclusively/predominantly breastfed infants or between exclusively and mixed breastfed infants within A-tetra- group. Nevertheless, as indicated in the main text, the results did not significantly differ whether 3-FL was included or excluded in the linear mixed effects model. Similarly, inclusion or exclusion of LNNT did not exhibit significant impact on the analyses results. Therefore, both 3-FL and LNNT were included in the models for consistency.

**Supplementary Table 1 Age- removed HMO concentrations between A-tetra+ and A-tetra- groups with the same feeding practices**

|  | Exclusive/ Predominant (EBF) | | | Mixed Breastfeeding (MBF) | | |
| --- | --- | --- | --- | --- | --- | --- |
| HMO  Concentrations | A-tetra+  (45 samples) | A-tetra-  (107 samples) | p-value | A-tetra+  (6 sample) | A-tetra-  (19 samples) | p-value |
| 2’-FL  residual | -1.62  (676.78) | 9.16  (942.21) | 0.94 | 269.72  (1128.87) | -3.39  (831.03) | 0.6 |
| 3-FL  residual | -297.93  (488.83) | 247.19  (1199.66) | 0 | 98.13  (927.04) | -195.47  (723.91) | 0.5 |
| 3’-SL  residual | -0.04  (0.45) | 0.05  (0.34) | 0.23 | -0.14  (0.38) | -0.01  (0.37) | 0.47 |
| 6’-SL  residual | 1.8  (42.34) | 0.04  (50.25) | 0.83 | -6.92  (10.86) | -1.16  (22.77) | 0.41 |
| LNT  residual | 12.59  (350.06) | -7.9  (239.8) | 0.72 | 26.64  (266.92) | -35.08  (167.76) | 0.61 |
| LNNT  residual | 10.33  (54.22) | -12.72  (49.44) | 0.02 | 34.71  (116.18) | 45.08  (96.6) | 0.85 |
| LNFP-I  residual | -39.61  (249.45) | 37.04  (279.17) | 0.1 | -41.15  (198.74) | -42.47  (139.44) | 0.99 |
| A-tetra  residual | -2.78  (83.16) | 0  (0) | 0.82 | 11.81  (148.38) | 0  (0) | 0.85 |

**Supplementary Table 2 Age- removed HMO concentrations between two feeding practices in either A-tetra+ or A-tetra- group**

|  | A-tetra+ | | | A-tetra- | | |
| --- | --- | --- | --- | --- | --- | --- |
| HMO  Concentrations | EBF  (45 samples) | MBF  (6 sample) | p-value | EBF  (107 samples) | MBF  (19 samples) | p-value |
| 2’-FL  residual | -1.62  (676.78) | 269.72  (1128.87) | 0.59 | 9.16  (942.21) | -3.39  (831.03) | 0.95 |
| 3-FL  residual | -297.93  (488.83) | 98.13  (927.04) | 0.35 | 247.19  (1199.66) | -195.47  (723.91) | 0.04 |
| 3’-SL  residual | -0.04  (0.45) | -0.14  (0.38) | 0.59 | 0.05  (0.34) | -0.01  (0.37) | 0.58 |
| 6’-SL  residual | 1.8  (42.34) | -6.92  (10.86) | 0.27 | 0.04  (50.25) | -1.16  (22.77) | 0.87 |
| LNT  residual | 12.59  (350.06) | 26.64  (266.92) | 0.91 | -7.9  (239.8) | -35.08  (167.76) | 0.55 |
| LNNT  residual | 10.33  (54.22) | 34.71  (116.18) | 0.63 | -12.72  (49.44) | 45.08  (96.6) | 0.02 |
| LNFP-I  residual | -39.61  (249.45) | -41.15  (198.74) | 0.99 | 37.04  (279.17) | -42.47  (139.44) | 0.06 |
| A-tetra  residual | -2.78  (83.16) | 11.81  (148.38) | 0.82 | 0  (0) | 0  (0) |  |

**Supplementary Information-3: Exploratory data analysis on 3’-SL**

Examining the distribution of 3’-SL with age, note that 3’-SL not only increases but also the variance becomes larger with age (**Supplementary Figure 2a**). The increased variance with age is even more apparent after regressing out the age effects (**Supplementary Figure 2b**). In order to minimize the effects of heteroscedasticity and fulfill linear assumption using linear mixed effects model, log-transformation was applied to 3’-SL (**Supplementary Figure 2c**). Notice the variance of age corrected 3’-SL is more consistent with age (**Supplementary Figure 2d**). Nonetheless, we have conducted association analyses using the original and log-transformed 3’-SL, separately and the conclusions of our main findings remain unchanged.


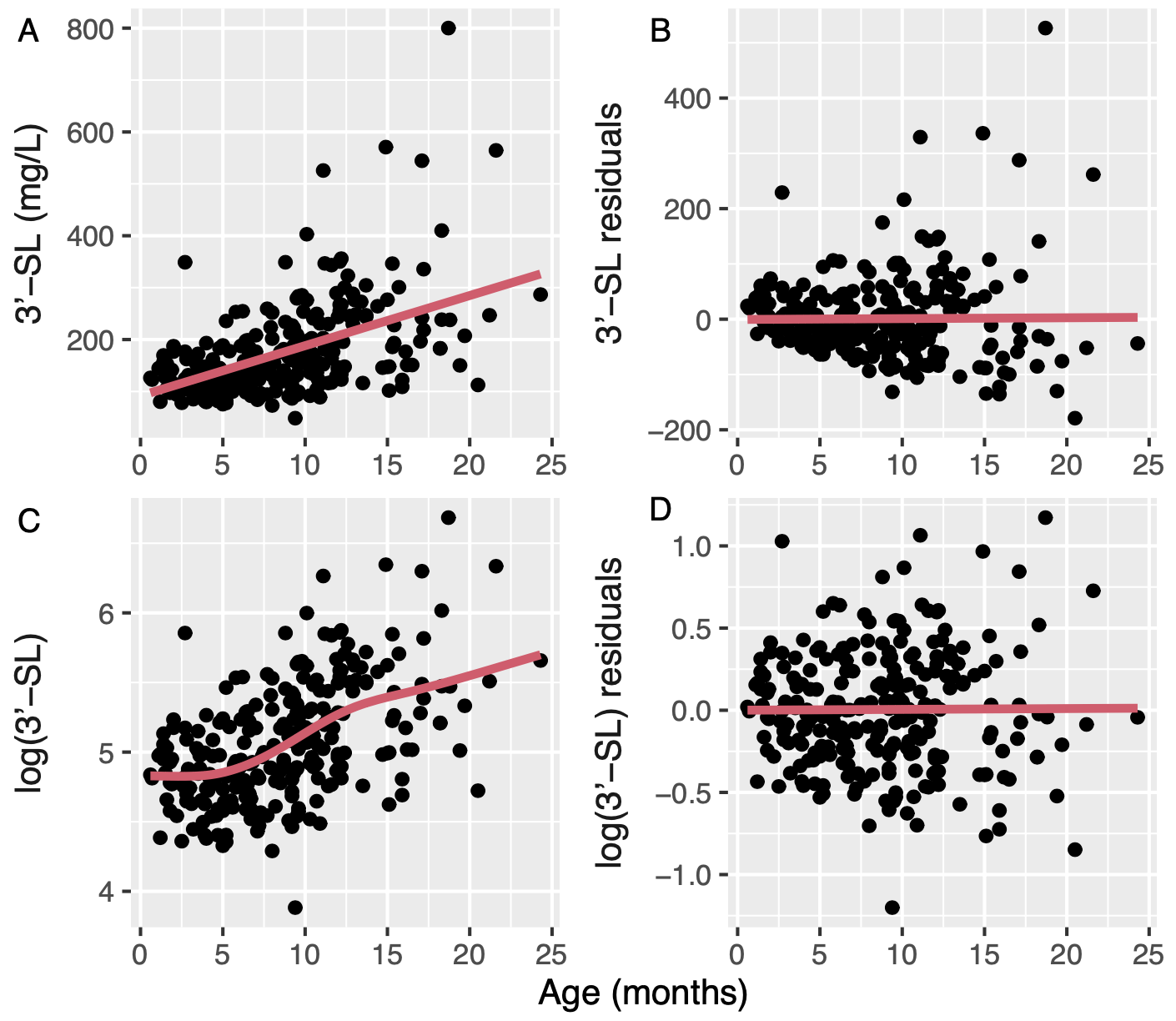


Supplementary Figure 2: Comparison of raw and age-removed 3’-SL with and without log-transformation. Each figure shows the relationship between age in months and the raw 3’-SL in mg/L (2a (top left)), age-removed 3’-SL (2b (top right)), log-transformed 3’-SL (2c (bottom left)), and age-removed log-transformed 3’-SL (2d (bottom right)). The x-axis represents the age in months.

As shown in **Figure 2** of the main text and **Supplementary Figure 2**, 3’-SL exhibits an increased concentration with age, underscoring the importance of removing age effects prior to conducting the association analyses. As indicated in the Subjects and Methods section, age effects for HMOs were removed prior to any analysis. The spaghetti plots of the raw 3’-SL (left) and age-removed 3’-SL (right) (**Supplementary Figure 3)**, respectively, are provided to discern the potential intra-subject variations of 3’-SL with age. While some individuals show a high intra-subject variation of 3’-SL concentration with age, the intra-subject variation diminishes after the age effects were removed. Since age-removed 3’-SL was used in the analyses and the linear mixed effects model fitted for the age-removed 3’-SL exhibited no significant association between age and 3’-SL, the intra-subject variation of 3’-SL concentrations with age would not affect the association results reported in our study.


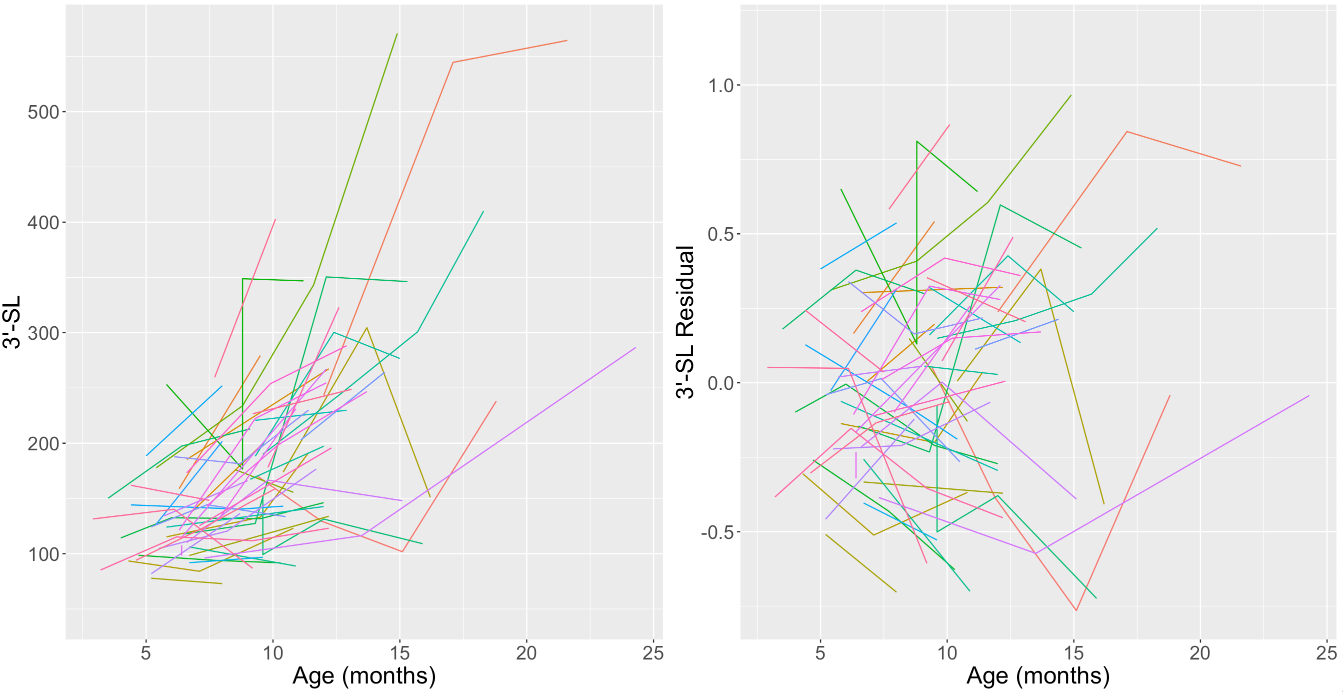


Supplementary Figure 3: The spaghetti plots of the raw 3’-SL (left) and age-removed 3’-SL (right) with age. Each connected line represents the concentration of 3’-SL of a given subject. The x-axis represents the age in months.

**Supplementary Information-4: Subgroup analyses for A-tetra+ and A-tetra- groups and Lewis blood groups**

Model 2 was employed in the main text to jointly evaluate the associations between MSEL and HMOs. Alternatively, it is feasible to evaluate the associations of A-tetra+ and A-tetra- groups with MSEL using Model 1, separately. When Model 1 was fitted to the A-tetra- subset, no significant association was observed between MSEL and HMOs, consistent with the reported main findings. For the A-tetra+ subset, significant association between age-removed 3’-SL and ELC score (p = 0.005, EF: 12.6) was observed. However, no association was observed between 3’-SL and the two language subdomain scores, although the adjusted p-value for the association with receptive language subdomain was 0.06. While these results are somewhat consistent with those obtained using Model 2, a single model (Model 2) comprising of all data has more statistical power and is more robust. When Model 1 was applied separately to the A-tetra+ and A-tetra- subjects, the sample size was reduced, resulting in less power. This could explain why no association was found between 3’-SL and receptive and expressive language scores among the A-tetra+ subjects. In addition, since only the samples with similar traits were used in subgroup analyses, the robustness of the results could be a subject of question. On the other hand, Model 2, allowing data with different traits to be fitted altogether, would be more robust.

Since genetic polymorphisms have been widely implicated to modulate human milk composition, we grouped our cohort into four Lewis blood groups based on 2’-FL and LNFP-II. 2’-FL and LNFP-II represent $\alpha$ 1,2- and $\alpha$ 1,4-fucosylated HMOs, respectively. Women with $\alpha$ 1,2-fucosylated structures were grouped as Se+ or otherwise, Se-. Women with $\alpha$ 1,4-fucosylated were grouped as Le+ or otherwise, Le-. The benchmark for Se- was 2’-FL < 15mg/L and for Le- was LNFP-II < 30mg/L (2). A given subject with at least one sample grouped as positive was considered in a positive cluster. The numbers of subjects and samples for each blood group and detectability of A-tetra are summarized in **Supplementary Table 3**. In our data, 144 samples from 76 subjects were in Se+Le+ group, 31 samples from 18 subjects in Se-Le+ group, 6 samples from 4 subjects in Se+Le- group, and 2 samples from one subject in Se-Le- group.

**Supplementary Table 3 Numbers of human milk samples^1^**

|  | Se+Le+ | Se-Le+ | Se+Le- | Se-Le- |
| --- | --- | --- | --- | --- |
| A-tetra+ | 56 (32) | 0 | 1 (1) | 0 |
| A-tetra- | 88 (44) | 31 (18) | 5 (3) | 2 (1) |
| Total | 144 (76) | 31 (18) | 6 (4) | 2 (1) |

^1^ Numbers of subjects are in the parentheses.

With the limited sample sizes, Model 1 was not fitted to Se-Le- and Se+Le- groups. For Se-Le+ group, there was no significant relationship between MSEL and HMOs. For Se+Le+ group, where the sample size was the largest, significant relationships between 3’-SL and ELC score (p=0.02) and receptive language (adjusted p=0.045) were observed with effect sizes 6.95 and 6.41, respectively. The above findings appear to suggest that the associations between 3’-SL and ELC and receptive language in the Se+Le+ group may be independent of the A-tetra status. To further address this question, Model 2 was applied to the Se+Le+ group where stratification of subjects based on the A-tetra status was employed. Significant associations of 3’-SL with ELC (p=0.001, EF: 12.93), receptive (adjusted p=0.015, EF: 9.82) and expressive language scores (adjusted p=0.016, and EF: 8.13), respectively, were observed in the A-tetra+ group. Therefore, the positive associations observed using Model 1 are most likely driven mainly by the A-tetra+ subjects. Nevertheless, given the limited sample sizes in the Se-Le- and Se+Le- groups, it is difficult to definitively conclude whether or not the associations between HMOs and early cognitive development depend on the Lewis blood group. Studies with larger and balanced sample sizes among the four Lewis blood groups are warranted to better understand the implications of genetic polymorphisms and early cognitive development.

**Supplementary Information-5: Maltotriose quantitative HMOs analysis**

Maltotriose standard curves were used to quantify HMOs consisting of 24 different types, including the aforementioned eight HMOs, plus 3’-galactosyllactose (3’-GL), 6’-galactosyllactose (6’-GL), lactodifucotetraose (LDFT), Lacto-N-fucopentaose II (LNFP-II), Lacto-N-fucopentaose III (LNFP-III), Lacto-N-fucopentaose V (LNFP-V), Lacto-N-neofucosylpentaose (LNnFP), Lacto-N-difucohexaose-I (LNDFH-I), Lacto-N-neodifucosylhexaose (LNnDFH), sialyl-lacto-N-tetraose b (LST b), sialyl-lacto-N-tetraose c (LST c), difucosyl-lacto-N-hexaose a (DFLNHa), Disialyl-lacto-N-tetraose (DSLNT), N-acetylneuraminic acid (Hex4 HexNAC2) (1), Hex4 HexNAC2 (2), and monofucosyllacto-N-hexaose-III (MFLNH-III). Given the limited sample size of our study, exploratory analyses were conducted using the 24 Maltotriose quantitative HMOs using Model 2. With an increased number of variables, variable selection using backward elimination was applied to reduce the number of variables. The two random intercepts, the site, batch and the indicator for the A-tetra- variables were not subjects of variable selection and were always included in the models. That is, only the 24 HMOs were considered for variable selection. In addition, since the sample size was limited and the covariates used in the models for each MSEL score were different, the p-values shown below were not corrected for multiple comparisons.

For the ELC and receptive language scores, the final model using the backward elimination showed a significant positive association with age-removed 3’-SL (both p < 0.001) and a negative association with 3’-GL (p = 0.001 and p = 0.002, respectively). This finding further supports the main analysis results. In addition, gross motor had a significant positive association with age-removed LST c (p < 0.001) and significant negative associations with 3’-SL (p = 0.042) and LNFP-V (p < 0.001), respectively. Visual reception had significant positive associations with 3-FL (p < 0.001), 6’-GL (p < 0.001), 3’-SL (p = 0.002), LNNT (p = 0.004), LNDFH-I (p = 0.006), LST c (p = 0.028), and MFLNH-III (p = 0.039) and negative associations with age-removed LDFT (p < 0.001), LNNFP (p = 0.019), LST b (p < 0.001), Hex4 HexNAC2 (p < 0.001) (1), and A-tetra (p = 0.047), respectively. Expressive language had a significant positive association with 3’-SL (p = 0.01).

While the above results suggested marginal significant associations among some of the 24 HMOs and MSEL, with the limited sample sizes, these associations were no longer significant after correcting for multiple comparisons. Future studies with a larger sample size will be needed to further shed light on the potential associations among these HMOs and MSEL.

**Supplementary Information-6: Study limitations**

Our study consists of several potential limitations. First, the time taken for the acquisition of human milk sample after the last feeding nor the frequency of breastfeeding was collected, which led to the potential concerns of dose effects. Nevertheless, our study is an observational study without any intervention where subjects meeting the inclusion and exclusion criteria were enrolled, minimizing selection biases. Furthermore, as shown in **Table 1**, the demographics, the SES and maternal factors are statistically similar between the two groups. In addition, a previous study has shown a limited variability in daily weight-normalized human milk intake in healthy term infants among the reported studies, suggesting that the probability of the observed results being due to an alteration of milk intake is low (3). Therefore, the dose effects should be similar between the A-tetra+ and A-tetra- groups and are unlikely to contribute to the observed results.

Second, other than the SES and maternal factors, there are other potential factors that might modulate the children cognitive abilities. Since the first language spoken at home could potentially affect children’s language ability, it warrants additional examinations on the first language spoken at home. Of the 99 subjects, the language spoken at home was available from 82 participants (82.8%). Among the 82 participants, English was the first language spoken at home for 79 participants (96.3%) and the Japanese, Arabic, and Spanish were the first languages spoken at each home for the remaining three subjects. The Mullen receptive and expressive language t-scores of these subjects were 46, 51, and 57 for receptive and 48, 55, and 64 for expressive language t-scores, respectively, which are all within the normal ranges. The distribution of the first language spoken at home for A-tetra+ and A-tetra- groups are provided in **Supplementary Table 4**.

**Supplementary Table 4 Distribution of the first language spoken at home**

|  | A-tetra+ | A-tetra- |
| --- | --- | --- |
| English | 25 | 54 |
| Other | 0 | 3 |
| Missing information | 8 | 9 |

We further evaluated the multiple birth information, which was collected from 91 subjects (91.9%) who were all singletons whereas information was not collected from the remaining 8 subjects (**Supplementary Table 5**).

**Supplementary Table 5 Distribution of multiple birth status**

|  | A-tetra+ | A-tetra- |
| --- | --- | --- |
| Singletons | 28 | 63 |
| Multiple birth | 0 | 0 |
| Missing information | 5 | 3 |

Maternal parity (**Supplementary Table 6**) and race (**Supplementary Table 7**) were also taken into consideration as the potential factors. The parity was calculated as the number of gravidity, the number of times a woman is or has been pregnant regardless of the pregnancy outcome, minus the number of abortions. Nevertheless, unlike the information of race, which is available for all subjects, it should be noted that the parity information was collected from 44 out of the 99 participants (44.4%).

**Supplementary Table 6 Distribution of the number of maternal parity**

| Number of parity | A-tetra+ | A-tetra- |
| --- | --- | --- |
| 1 | 7 | 11 |
| 2 | 7 | 11 |
| 3 | 0 | 3 |
| 4 | 1 | 0 |
| 5 | 1 | 2 |
| 6 | 0 | 0 |
| 7 | 0 | 1 |

**Supplementary Table 7 Distribution of race**

| Race | A-tetra+ | A-tetra- |
| --- | --- | --- |
| White | 32 | 55 |
| Asian | 0 | 5 |
| Black / African American | 0 | 3 |
| Pacific Islander | 0 | 1 |
| More than one race | 1 | 2 |

The distributions of the number of maternal parity and race were not significantly different between the two groups. Together with the statistically identical SES and maternal factors between the A-tetra+ and A-tetra- groups (**Table 1**), the contributions of the potential confounding factors to the observed results should be minimal. Thus, these factors were not included in the analysis. Moreover, most of the subjects being White, English speaking in high SES families lacked in diversity. Further studies with more diverse demographics and SES will be needed for more general interpretations.

Third, the examiners for administering MSEL were not blinded to the subjects’ feeding practices. Fourth, an accelerated longitudinal design was employed, making it difficult to conduct prediction analyses. Also, the data was imbalanced; each subject measured at different times and having different number of visits. While the employed model can manage the above limitations, it resulted in a smaller power for the analyses. In addition, two mothers enrolled siblings into the study. The employed statistical models did not account for the dependencies among these siblings. However, we compared the results without considering the dependence of siblings to that including the mothers as random intercepts. No significant change was observed. Fifth, with the mean age of our cohort being about 10 months, the insufficient age coverage might have not been sufficient to uncover the age interaction of 3’-SL and expressive language. In addition, while we have observed positive associations between 3’-SL concentrations and language development in the A-tetra+ group, the biological links between 3’-SL and language development remains elusive. Future studies focusing on this area are warranted. Finally, the current study investigated specifically the role of oligosaccharides on cognitive function development. Since other milk components, such as fatty acids, were not investigated, possibility of some common genetic variance mediated via other milk components (such as fatty acid milk composition altered in association with common genetic variations in fatty acid desaturase-2) cannot be excluded.

**References**

1. Howell BR, Styner MA, Gao W, Yap P-T, Wang L, Baluyot K, Yacoub E, Chen G, Potts T, Salzwedel A, et al. The UNC/UMN Baby Connectome Project (BCP): An overview of the study design and protocol development. NeuroImage. 2019;185:891–905.

2. Wang M, Zhao Z, Zhao A, Zhang J, Wu W, Ren Z, Wang P, Zhang Y. Neutral Human Milk Oligosaccharides Are Associated with Multiple Fixed and Modifiable Maternal and Infant Characteristics. Nutrients. MDPI; 2020;12:826.

3. Yeung CHT, Fong S, Malik PRV, Edginton AN. Quantifying breast milk intake by term and preterm infants for input into paediatric physiologically based pharmacokinetic models. Matern Child Nutr. John Wiley & Sons, Ltd; 2020;16:e12938.
